# Modelling photovoltaic soiling losses through optical characterization

**DOI:** 10.1038/s41598-019-56868-z

**Published:** 2020-01-09

**Authors:** Greg P. Smestad, Thomas A. Germer, Hameed Alrashidi, Eduardo F. Fernández, Sumon Dey, Honey Brahma, Nabin Sarmah, Aritra Ghosh, Nazmi Sellami, Ibrahim A. I. Hassan, Mai Desouky, Amal Kasry, Bala Pesala, Senthilarasu Sundaram, Florencia Almonacid, K. S. Reddy, Tapas K. Mallick, Leonardo Micheli

**Affiliations:** 1 Sol Ideas Technology Development, P.O. Box 5729, San José, California 95150 USA; 2 National Institute of Standards and Technology, 100 Bureau Drive, Gaithersburg, MD USA; 3 University of Exeter, Penryn, UK; 4 Centro de Estudios Avanzados en Energía y Medio Ambiente (CEAEMA), Universidad de Jaén, 23071 Jaén, Spain; 5 Academy of Scientific and Innovative Research, Chennai, 600113 India; 6 Tezpur University, Tezpur, India; 7 Heriot-Watt University, Dubai, UAE; 8 Robert Gordon University, Aberdeen, UK; 9 Department of Chemistry, South Valley University, 83523 Qena, Egypt; 10 Faculty of Environmental Studies, University of York, Toronto, M3J 1P3 ON Canada; 11 Nanotechnology Research Centre, The British University in Egypt, 11837 El Sherouk City, Cairo, Egypt; 12 CSIR-Central Electronics Engineering Research Institute & Academy of Scientific and Innovative Research, Chennai, 600113 India; 13 Indian Institute of Technology Madras, Chennai, India; 14 National Renewable Energy Laboratory, Golden, CO USA

**Keywords:** Energy science and technology, Physics, Electronics, photonics and device physics, Optical physics, Techniques and instrumentation, Optical physics, Optics and photonics, Optical techniques, Imaging and sensing, Microscopy, Optical spectroscopy

## Abstract

The accumulation of soiling on photovoltaic (PV) modules affects PV systems worldwide. Soiling consists of mineral dust, soot particles, aerosols, pollen, fungi and/or other contaminants that deposit on the surface of PV modules. Soiling absorbs, scatters, and reflects a fraction of the incoming sunlight, reducing the intensity that reaches the active part of the solar cell. Here, we report on the comparison of naturally accumulated soiling on coupons of PV glass soiled at seven locations worldwide. The spectral hemispherical transmittance was measured. It was found that natural soiling disproportionately impacts the blue and ultraviolet (UV) portions of the spectrum compared to the visible and infrared (IR). Also, the general shape of the transmittance spectra was similar at all the studied sites and could adequately be described by a modified form of the Ångström turbidity equation. In addition, the distribution of particles sizes was found to follow the IEST-STD-CC 1246E cleanliness standard. The fractional coverage of the glass surface by particles could be determined directly or indirectly and, as expected, has a linear correlation with the transmittance. It thus becomes feasible to estimate the optical consequences of the soiling of PV modules from the particle size distribution and the cleanliness value.

## Introduction

Soiling has a negative impact on the economic revenues of PV installations, not only because it reduces the amount of energy converted by the PV modules, but also because it introduces additional operating and maintenance costs and, at the same time, increases the uncertainty on the estimation of PV performance, leading to both higher financial risks and interest rates charged to plant developers. Power reductions greater than 50% have been reported in the literature because of soiling^1,2^; it has been estimated that an average loss of 4% on the global annual energy yield of PV could cause losses in revenue on the order of 2 × 10^9^ US$ annually^3^.

A careful monitoring of soiling is required to mitigate its effect^4^. Soiling losses are generally quantified by using soiling stations. These systems are made of at least two PV devices, one of which is regularly cleaned while the other is left to soil naturally. By comparing the ratio of the electrical outputs of the two devices, it is possible to estimate the impact of soiling on the PV performance^5,6^. The International Electrotechnical Commission’s (IEC) metric to monitor and quantify the impact of soiling on PV modules is the soiling ratio, *r*_*s*_, which expresses the ratio of the electrical output of a soiled PV device to the output of the same device under clean conditions^7^. Like the transmittance, a higher soiling ratio translates to less soiling deposited on the modules. A value of 1 indicates clean conditions, with no soiling. For a more detailed definition of *r*_*s*_, please refer to the Methodology section. The fractional loss of solar-generated power due to soiling is 1 − *r*_*s*_.

Soiling stations have the advantage of directly measuring the impact of soiling on PV, but require careful maintenance to avoid significant measurement errors^8^. Novel sensors that require less maintenance and do not need a clean reference PV device are getting the attention of the market, and are based on the optical characterization of a soiled glass coupon^9,10^. Recently, a new procedure to estimate soiling losses using transmittance data has been validated through a one year study in the south of Spain^11^. Rather than using soiling stations or sensors, there are also methods to extract losses directly from PV performance data^12,13^.

All these methods directly measure the impact of soiling or estimate the losses of PV power based on the broadband transmittance of a soiled glass plate. On the other hand, they do not consider the optical properties, the composition, and the size distribution of particles deposited via soiling. These factors can have an impact on the adhesion of soiling to the PV module’s surface and need to be investigated if effective soiling mitigation strategies, such as cleaning methods or anti-soiling coatings, are going to be developed.

Previous studies^14,15^ investigated the optical, chemical and mechanical characteristics of dust particles collected on PV modules installed in the Middle East. Despite the importance of these studies toward the characterization of dust in high-soiling regions, they did not investigate the fundamental connections between the properties of the dust and the PV losses. Prior studies on the spectral characteristics of the optical transmittance and the particle size distribution of soiled glass coupons have been reported^16^, but not for natural soiling. In a separate study, a variety of artificial soil types were sprayed onto glass to study the resulting optical properties and PV panel spectral quantum efficiency^17^. In contrast to artificial soiling, other groups measured the transmittance loss versus the mass of deposits accumulated on glass plates soiled outdoors, also taking note of the angle of incidence of the light^18^. Borrowing from prior studies in atmospheric science, dry deposition rates on surfaces, aerosol optical properties, and Mie scattering theory, researchers have also developed a parameter to determine the change in PV panel transmittance for a given mass per unit area of atmospheric particulate matter (PM) deposited^19^. The experimental work utilized deposits collected from soiled PV panels in Gandhinagar, India, and took into consideration both particle absorption and scattering from a variety of particles, such as soot, salts, pollen, and mineral dust. Separate tests carried out outdoors in Mumbai, India, measured spectral reflectance, angle of incidence effects, and quantum efficiency for carefully modified full-sized PV modules^20^. All of these investigations of natural soiling gave important contributions to the field, but were conducted at only one location, and did not compare the optical properties of PV glass soiled at multiple sites with diverse climate conditions.

This work presents the results of an international collaboration that investigated the spectral effects of soiling naturally deposited on PV glass installed at various locations worldwide. Outdoor tests have been conducted at seven locations worldwide: Golden (Colorado, USA), San José (California, USA), Chennai (India), Jaén (Spain), El Shorouk City (Egypt), Tezpur (India), and Penryn (UK). These places were chosen to represent a wide variety of climates and environmental conditions (See the locations listed in Supplementary Information)^21–24^. Identical low-iron glass coupons were soiled at each location over an 8-week period between January and March 2016^25^. The samples were mounted horizontally (i.e., without a tilt). Because of the short data collection period, and of the single measurement taken only at the end of it, the soiling is not expected to be wholly representative of the sites. Correlating soiling with the specific climate conditions of a site is outside the scope of this work, which aims to investigate the spectral and optical characteristics of different types of soiling to attempt to find commonalities. The spectral transmittance and the particle size distribution (PSD) of the soiling was compared in order to find correlations that could be universally valid, and that could open possibilities to modelling PV soiling losses through the optical characterization of dust.

The present work draws upon one of the largest pools of naturally deposited soiling samples. While most of the soiling studies focus on a single site, there are only a limited number of investigations conducted on soiling from multiple locations. Together with this, we present the first effort where empirical models from other disciplines are brought in to describe both the spectral and optical characteristics of the samples, together with an examination of their corresponding PSDs.

**Figure 1:**
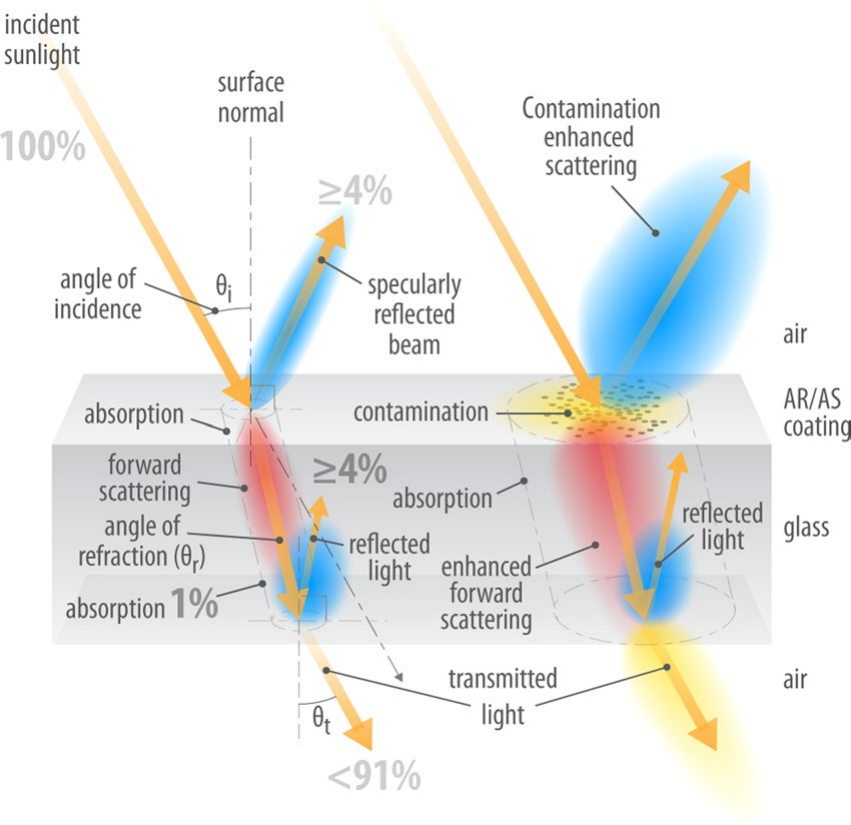
Effect of soiling on the transmittance and reflectance of light incident on glass. Diagram courtesy of Al Hicks/NREL and used by permission.

Realizing the complexities of a commercial PV module from the optical standpoint, the system under study in the present report was simplified so that it just includes the soiling on a sheet of low-iron glass (see Fig. 1). In this case, the light propagates through the glass into air again and can be detected. The left hand side of Fig. 1 shows the case where there is negligible soiling on top of the glass. Light is incident on the front of the glass at an angle *θ*_i_, as measured from the surface normal. This light is reflected and transmitted in accordance with Fresnel’s equations. As a rule of thumb, 4% of the light is reflected at each air-glass interface at near-normal incidence. Here, the subscripts i, r, and t denote incident, refracted and transmitted light, respectively. Light that is transmitted at the air-glass interface is refracted via Snell’s law and can reach the interface at the back to be transmitted out of the glass (i.e., via the second glass-air interface). Less than 91% of the incident light exits the glass (at θ_i_ = θ_t_) for uncoated, clean glass.

Contamination and soiling shown on the right hand side of Fig. 1 complicates the propagation of light via enhanced forward and backward scattering, as well as reflection and absorption. For each component, there is an angular spreading of the beam. Scattering, in the simplest case, can be understood from Mie theory, applicable for homogeneous spheres. However, in general, the irregular shapes and internal structure of naturally-occurring particles results in much more complicated scattering behavior. In addition, it is well known that *r*_*s*_ is a function of the angle of the incoming light^18,20^. Indeed, this is why it is defined in the IEC standard for conditions found at noon^7^.

Also shown in Fig. 1 is the possibility that glass utilized for commercial PV and solar thermal applications can often have an anti-reflection (AR) coating at the front air-glass interface. These coatings are typically a graded porous silica layer that presents a gradual increase of the index of refraction, n, from its value in air to the bulk glass (n = 1.49). They are currently being explored for additional properties that they might confer as anti-soiling (AS) or self-cleaning coatings^1–3^. In this study, we utilized bare glass without any coatings.

## Results and Discussion

Spectral hemispherical transmittance was measured for each of the soiled glass coupon samples. As explained in the Methodology, all the transmittance values mentioned in this work are relative hemispherical transmittance, in that they are the ratio of the transmittance of the soiled coupon to the transmittance of a clean reference coupon. The soiled coupons represent a snapshot in time for a particular location and are affected by the specific weather events that occurred during the soiling period^11^. Measurements were performed at two locations on each sample and the results were analyzed as described below. Table 1 summarizes the optical characteristics of the soiling collected after 8 weeks at the locations of the study. The first column represents the broadband relative transmittance for the wavelength range 350 nm to 1100 nm relevant to photovoltaic conversion. This is a simple average of the hemispherical relative transmittance values over that wavelength range. Also reported is the solar-weighted relative transmittance, calculated according to prior studies that utilize it to study the changes in the optical properties of polymer materials that encapsulate the solar cells^26^. It should be noted that the relative transmittance of soiling is not necessarily equivalent to a soiling ratio for a PV module. Indeed, the numerator of the soiling ratio (*r*_*s*_) for a PV module is the integral over wavelength of the product of the relative transmittance due to the soiling, the spectral response of the solar cell, *SR*(*λ*), and the incoming solar spectral irradiance^11^ (For a graphical representation of each of these terms, please refer to Supplementary Information). The denominator for *r*_*s*_ is a similar integral, but omits the transmittance. The resulting predicted soiling ratios are also reported in Table 1. These have been calculated following the previously described procedure utilizing the standard air mass 1.5 (AM1.5) solar irradiance spectrum^11,27^ and summarized in the Methods section. For the majority of the sites, the three approaches yield very similar values.

**Table 1:** Soiling relative transmittance (τ_b_), solar weighted transmittance and predicted soiling ratio (*r*_*s*_) for a monocrystalline silicon cell at the indicated sites.

City	Broadband Relative Transmittance (τ_b_)	Solar Weighted Transmittance	Predicted Soiling Ratio (r_s_)
Chennai, India	0.907	0.904	0.909
El Shorouk, Egypt	0.670	0.659	0.674
Golden (CO), USA	0.970	0.969	0.970
Jaén, Spain	0.943	0.941	0.945
Penryn, UK	0.996	0.995	0.996
San José (CA), USA	0.982	0.980	0.982
Tezpur, India	0.976	0.975	0.977

The wavelength range was from 350 nm to 1100 nm. The relative transmittance is obtained as the ratio of the transmittance of each soiled glass to the transmittance of the clean reference glass. The standard uncertainty associated with the reproducibility of the measurements is estimated to be ±0.005. The values for each site have been obtained as average of the individual measurements shown in Table S2.

### Spectral transmittance and the Ångström equation

**Figure 2:**
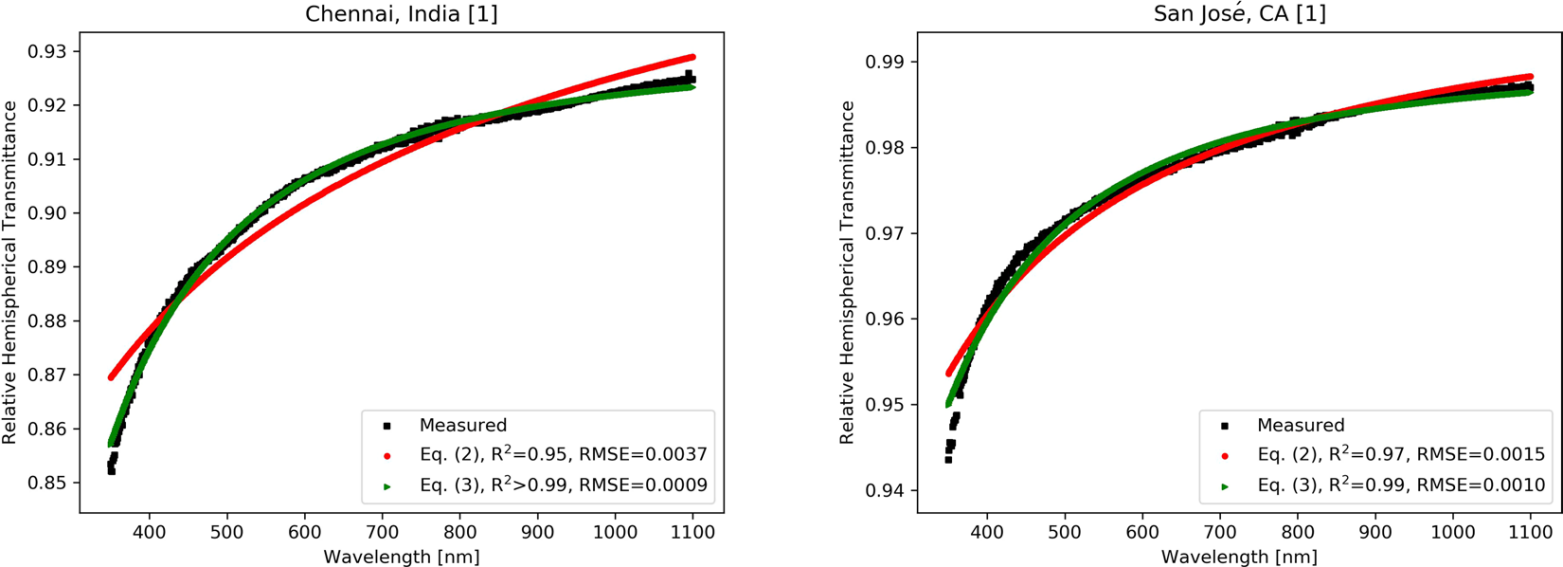
Transmittance vs. wavelength (350–1100 nm) curves for glass coupons soiled at two representative locations, (left) Chennai, India, and (right) San José, California. Also shown is the fit to the modified Ångström equations, (red) Eq. (2) and (green) Eq. (3). The measurements are referenced to clean glass.

Fig. 2 shows the transmittance vs. wavelength data for glass coupons soiled at two representative locations, Chennai, India, and San José, California. The number in brackets (1 or 2) after the site name indicates the spot on the glass coupon. In general, the curves of the hemispherical transmittance versus wavelength for glass soiled at the seven sites do not have a completely flat profile, but rather they gradually rise with wavelength (For data at other locations, see Supplementary Information and the left side of Fig. S3). The higher losses due to soiling are found in the shorter wavelength regions where there is a lower spectral response for a crystalline silicon (c-Si) solar cell. This partially mitigates the deleterious effects on the predicted soiling ratio, *r*_*s*_, reported in Table 1. In general, however, one should not expect that the broadband transmittance values in that table to be equal to the soiling ratios for a given site. A complete overview on the impact of soiling depends on the PV technology (e.g., the semiconducting PV absorber materials), the location of the PV module, and the amount of time that has passed since the module was cleaned^11^.

There are additional reasons that make the results of Fig. 2 noteworthy. Polymers, such as ethylene vinyl acetate, EVA, used to encapsulate the solar cells and bond them to the PV glass, are susceptible to yellowing when exposed to UV radiation, the extent of which depends on the dose and other environmental conditions^28^. The expected yellowing and degradation rates due to the outdoor UV exposure of polymer encapsulants and polymer back sheets in PV modules may therefore have to be adjusted if soiling is present.

The Ångström turbidity equation describes the attenuation of light by aerosols suspended in the atmosphere^29^. This turbidity is due to optical scattering that is primarily described by Mie theory, averaged over the distribution of particle sizes and optical properties. According to the Ångström turbidity equation, the transmittance *τ* of a column of air at a wavelength *λ* can be empirically modeled as,

1 $$\tau (\lambda )=\exp (\,-\,\beta \cdot {\lambda }^{-\alpha }\cdot m)$$

where *α* is an index relating to the size of the particles, *β* is a parameter representing the amount or concentration of aerosols, and *m* is the optical path length (typically, the air mass). The term *β* is typically expressed in units related to the number of particles per volume or by the mass of suspended material per volume^30^. It should be noted that the Ångström turbidity equation attempts to account for both scattering and absorption by the particles^29,30^. The value of *α* would be 4 for very small particles and 0 for very large particles. Distributions of particle sizes lead to intermediate values of *α*.

In order to consider the transmittance of aerosols and particles on the glass cover of a PV module, it is useful to modify the Ångström equation so that it is applicable to surfaces instead of volumes. We propose that its empirical approach can be modified to describe the wavelength dependence of the transmittance of light due to small particles adhering to the glass. These are some of the same particles that were originally suspended in the air. The product $\beta \cdot m$ can be combined into a new term, *β*_sur_, where the subscript “sur” denotes surface, which represents both the mass of particles per unit area on the glass surface, and the strength of forward scattering of those particles. Thus, we propose a reformulated version of the original Ångström turbidity equation,

2 $$\tau (\lambda )=\exp (\,-\,{\beta }_{{\rm{sur}}}\cdot {\lambda }^{-a}).$$

In order to better fit our data, we also found it useful to introduce an additional correcting offset parameter, γ*. This is a wavelength independent component due to very large particles that cannot stay suspended in the air, and hence fall on the glass surface. The fully-modified equation becomes,

3 $$\tau (\lambda )=\exp (\,-\,{\beta }_{{\rm{sur}}}^{\ast }\cdot {\lambda }^{-{a}^{\ast }}\,)+{\gamma }^{\ast }$$

This γ^*^ term can also correct for a small amount systematic errors due to light that goes undetected by the measurement system. Equations (1–3) utilize a relatively simple equation in a similar way as is done by the Sandia model for PV module performance in the field^31^. This set of largely empirical equations is used by PV practitioners and engineers to correct PV module performance from standard test conditions, for example, at a given module temperature and solar spectral irradiance, to those found in actual field operation. Such an approach was used to correct for angle of incidence effects for soiled PV modules^20^. The transmittance as a function of wavelength given by Eq. (3) can be analogously used to correct the PV performance for soiling for a given input solar spectral irradiance and a specific type of PV technology, as described in the methodology.

The values for the fit to the data using the modified Ångström turbidity equation are found in Table 2 for all of the sites. Since the coupons exhibited some non-uniformity in the soiling, two spots on each coupon were sampled, and three transmittance measurements for each spot were averaged. The two exceptions were Chennai and Jaén for which only one spot was averaged. These measurements represent a single snapshot in time, for the 8 week soiling period, for a given location, with the weather patterns that existed during that period. As it can be seen in Table 2, Eq. (3) always outperforms Eq. (2), achieving higher values of correlation coefficient *R*^2^ and lower root mean square error (RMSE). Plots of the fits to Eqs. (2) and (3) are shown in Fig. 2 for two representative locations, while Supplementary Information contains the data for all the locations.

**Table 2:** Results of the modified Ångström turbidity equation fits for wavelengths of 350 nm to 1100 nm.

City	α	α*	β_sur_	β_sur_*	γ*	R^2^ Eq. (2)	R^2^ Eq. (3)	RMSE Eq. (2)	RMSE Eq. (3)
Chennai, India	0.560	2.093	0.078	0.008	−0.070	0.951	0.997	0.0037	0.0009
El Shorouk, Egypt	0.616	2.132	0.314	0.029	−0.252	0.945	0.998	0.0128	0.0027
	0.621	2.073	0.315	0.032	−0.250	0.947	0.996	0.0127	0.0033
Golden (CO), USA	0.584	1.994	0.028	0.004	−0.025	0.916	0.950	0.0010	0.0007
	0.636	1.718	0.020	0.004	−0.017	0.924	0.933	0.0008	0.0008
Jaén, Spain	0.841	2.604	0.041	0.005	−0.040	0.940	0.998	0.0038	0.0007
Penryn, UK	2.103	3.889	0.002	0.000	−0.002	0.948	0.984	0.0015	0.0008
	2.092	2.988	0.001	0.001	−0.001	0.957	0.979	0.0011	0.0008
San José (CA), USA	1.219	2.098	0.013	0.005	−0.010	0.974	0.987	0.0015	0.0010
	1.447	1.904	0.008	0.004	−0.004	0.975	0.979	0.0011	0.0010
Tezpur, India	0.722	2.358	0.017	0.002	−0.016	0.937	0.983	0.0014	0.0007
	0.712	2.415	0.019	0.002	−0.018	0.936	0.988	0.0015	0.0006

**Figure 3:**
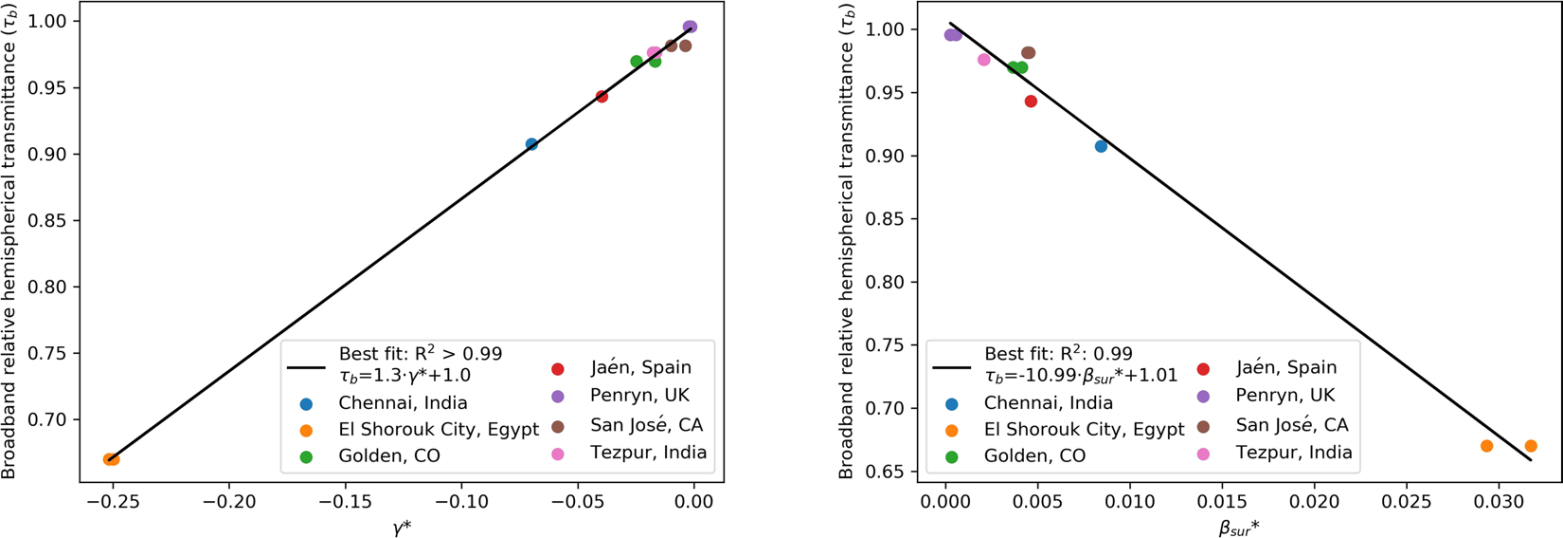
Broadband relative transmittance *τ*_b_ (350–1100 nm) as a function of γ^*^ (left-plot) and ${\beta }_{sur}^{\ast }$ (right-plot) for the seven study sites. The location is indicated by the color. All the sites except Jaén and Chennai had two spots measured on the coupon, and both were plotted separately.

Figure 3 shows the value of γ^*^ for the different coupons plotted against the broadband relative transmittance, *τ*_b_. In the case where two spots were probed, each data point was included separately. A linear relationship between γ^*^ and *τ*_b_ can be observed. A linear regression yields *R*^2^ > 0.99 and is given by:

4 $${\tau }_{b}=1.00\,+1.30\cdot {\gamma }^{\ast }$$

Taking the average value of Eq. (3) by integration over wavelength, one would expect a linear component proportional to γ^*^. That all seven sites should fall on the same line was somewhat unexpected. This finding could indicate that the absorption and scattering properties of many different types of particulate materials (i.e., mineral dust, soot, and pollen) found at the various sites are such that γ^*^ is paired with α^*^ and β^*^_sur_ in a way that leads to the linear relationship, Eq. (4). In this light, a linear relation with R^2^ of 0.99 is also found between β^*^_sur_ and the broadband hemispherical transmittance (right plot in Fig. 3). No correlation is found instead between α^*^ and the broadband hemispherical transmittance. The reason behind this might rely on the fact that, in accordance with the original formulation, shown in Eq. (2), the parameter α^*^ could be a function of the size of the particles.

Qasam and co-workers have studied the spectral characteristics of glass coupons soiled outdoors in Kuwait^16^. The results closely matched those obtained using a Mie scattering model. *SR*(*λ*), and the soiling ratio for a soiled PV module were also considered. Burton *et al*.^17^ used artificial soiling to explore soiling’s effect on a module’s quantum efficiency, which is directly related to *SR*(*λ*). That work established a strong correlation between the measurement of the spectral transmittance of glass coupons and quantum efficiency measurements for a module. The work of Piedra *et al*.^32,33^ considered the particle size distribution for the deposits and also utilized Mie scattering theory. Our experimental results strongly suggest that the general shape of the spectral curves found in each of these prior studies is also found under a diverse set of natural soiling conditions. In addition, we assert that the spectral transmittance  curves show a reasonable fit to an empirical model inspired by the Ångström turbidity equation, which also has at its roots Mie scattering theory.

### Particle size distribution

The optical properties of the soiled PV glass are expected to depend upon the size distribution of the deposited particles. That relationship, at first glance, may be challenging to find, given the range of sizes and shapes, their relative abundance and their differing chemical compositions. There are, however, a number of fundamental insights that can be gleaned from images obtained from a microscope. First, the open platform software ImageJ was used for image analysis of micrographs taken at 100× and 500× magnifications^34^ (For a complete description, please refer to the Methods section. For typical micrograph images, refer to the Supplemental Information). The image analysis attempted to identify each particle and its projected area, *A*, and the results were tabulated. The particle areas thus obtained were summed and the result was divided by the total analyzed area in the micrograph to yield the measured fractional area coverage, *f*.

**Figure 4:**
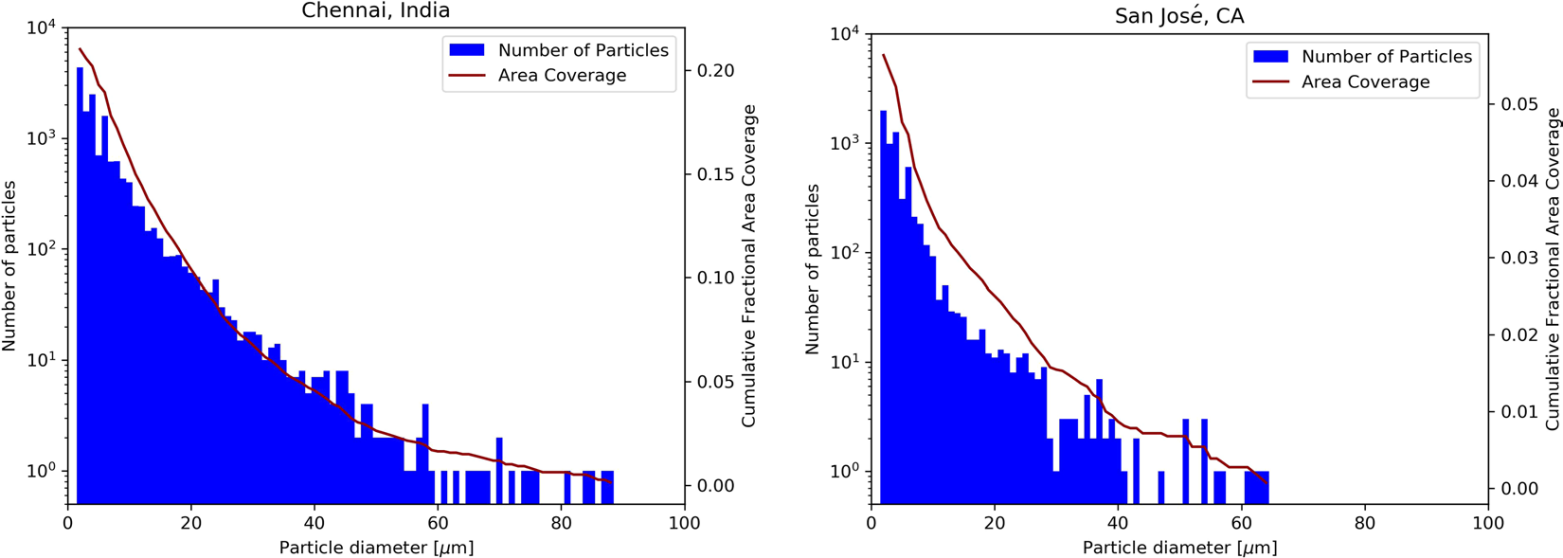
Particle size distribution density, *dN*(*D*)/*dD*, left y-axis, and cumulative fractional area coverage, on the right y-axis, both estimated by ImageJ using the 100× optical micrographs. Two representative locations are shown: (left) Chennai and (right) San José. The bin size is 1 µm.

The data was then further processed to estimate the particle size distribution density versus particle diameter, *D*. The effective diameter for an equivalent round particle was calculated from *D* = (4A/π)^1/2^. Some typical particle size distribution densities for coupons soiled outdoors are shown in Fig. 4 (left y-axis) for two representative locations, Chennai and San José (images taken at 100×). The cumulative fractional area coverage at a given value of *D* (summed from *D* to ∞) is shown on the right y-axis. This red line yields the same value of *f* that was previously mentioned as it approaches the smallest diameter values. It is apparent from the plot that one should not neglect the smallest of particles, in part because they are quite numerous and their combined coverage is significant. The shape of the curve for the number of particles and that for the particle area coverage appear the same even though the former is logarithmic, while the area coverage uses a linear scale.

Having obtained the particle size distribution density, we can then fit it to an existing model that may describe its shape or other properties. In the approach that follows, we exploited the framework described in the Institute of Environmental Sciences and Technology (IEST) Product Cleanliness Level standard^35^, IEST-STD-CC 1246E, to calculate the cleanliness level of each soiled coupon. The cumulative distribution of particle diameters is specified in this standard by a cleanliness level, *L*, with *N*(*D*) being the number of particles per unit area having a diameter between *D* and *L* (both in micrometers):

5 $$N(D)={10}^{0.926\cdot [{({\log }_{10}L)}^{2}-{({\log }_{10}D)}^{2}]}/(0.1\,{{\rm{m}}}^{2})$$

**Figure 5:**
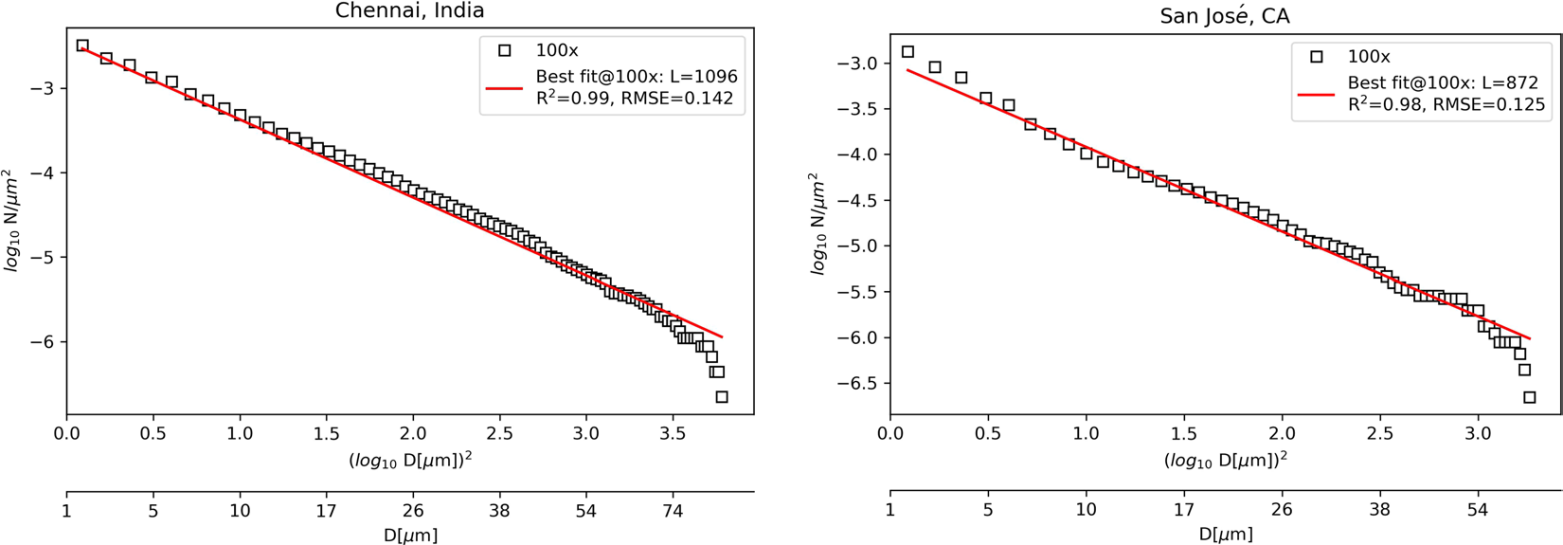
Cumulative particle size distribution and best fit (to IEST-STD-CC 1246E) at 100× for two representative locations.

Because one cannot take the logarithm of a number with the units, Eq. (5) implicitly has 1 µm divided into both *L* and *D*. For convenience, the cumulative distribution in the equation can also be expressed per unit square micrometer. The size distribution density for the particles is given by *dN*(*D*)/*dD*. Plotting log_10_ *N*(*D*) versus (log_10_ *D*)^2^ yields a straight line of slope 0.926 with a *y*-axis intercept that yields the cleanliness level, *L*. For a y-axis value on Fig. 5 close to −3, the corresponding *L* is approximately 870 µm, while –2.5 gives 1070 µm.

Figure 5 shows two representative plots (Others are given in Supplementary Information.). Though it is not well defined below 1 µm, the line was extended to data points less than that value. From this analysis, it was found that the particle size distribution for soiling on the glass coupons fits IEST-STD-CC 1246E with an *L* value lying within the range 600 to 1200 μm (Table 3). The lowest value is for Penryn, reflecting an extremely low level of soiling accumulating on the glass coupon due to frequent rain during the collection period. We repeated the procedure using each of the 100× and 500× images, calculating *R*^2^ and root-mean-square error (RMSE) in each case to quantify the goodness of the fit. Table 3 summarizes the results for all of the study sites and for the two different magnifications.

**Table 3:** Fit to the IEST-STD-CC 1246E cleanliness standard for each of the locations. The cleanliness level is given by *L*. Also indicated is the fractional area coverage *f* of the particles on the glass surface.

City	L at 100×	L at 500×	f at 100×	f at 500×	# of particles at 100×	# of particles at 500×	R^2^ at 100×	R^2^ at 500×	RMSE at 100×	RMSE at 500×
Chennai, India	1096	1154	0.21	0.25	14748	6794	0.99	0.98	0.142	0.110
Golden, CO	856	881	0.08	0.15	8512	9373	0.99	0.93	0.228	0.281
Jaén, Spain	1010	1145	0.16	0.26	19209	9445	0.99	0.93	0.130	0.238
Penryn, UK	684	670	0.02	0.06	2612	5845	0.86	0.79	0.372	0.385
San José, CA	872	1169	0.06	0.21	6116	7324	0.98	0.86	0.125	0.388
Tezpur, India	1167	1193	0.16	0.32	14423	8832	0.98	0.98	0.266	0.130

Like the results obtained by applying the Ångström turbidity equation, the particle size distribution and cleanliness standard findings highlight that there is a remarkable similarity between the seven sites in that they all fit the same general theoretical framework, despite the different amounts and types of soiling and diverse climate conditions. It was found that a magnification of 100× seems to yield better (higher *R*^2^) results than 500×, probably because the lower magnification covers 25 times as much area, yielding better statistics, and is less exposed to the effect of any non-uniformity of the soiling than the higher magnification, with the only exception for the coupon exposed in Tezpur, India (see Table 3). Using the lower magnification images also allows the consideration of larger particles in the analysis. Images at 500× magnification do suggest an abundance of small particles (<1 µm) (For micrographs, refer to Supplemental Information.).

Taken together, this suggests that future measurements of this type should employ a synergistic approach utilizing several magnification levels. Multiple images obtained as the sample is stepped in the *x* and *y* directions can be joined and combined as a mosaic for each magnification^36^. It should be noted that some care should be taken in the analysis used for calculating the cleanliness values, *L*, illustrated in Fig. 5, so as not to neglect either small or large particles. Failure to do this can skew the results and lead to errors in the estimated parameters.

### Fractional area coverage vs. cleanliness level

Ma, *et al*.^37^, and Perry^38^ recognized that the fractional area coverage *f* (also called the obscuration ratio) can be derived from the IEST-STD-CC 1246E distribution, yielding

6 $${\log }_{10}(f/ \% )=0.926\cdot {({\log }_{10}L)}^{2}-7.277$$

The 0.926 is the particle distribution slope given in Eq. (5). The equation is obtained by integrating the product of the size distribution density associated with Eq. (5) and the particle area *πD*^2^/4 with respect to *D*. (The intercept of 7.277 differs from that reported in previous work^37,38^, 7.245, because an older version of the cleanliness standard distribution, MIL-STD-1246B, for which the denominator was one square foot rather than 0.1 m^2^, was used.)

**Figure 6:**
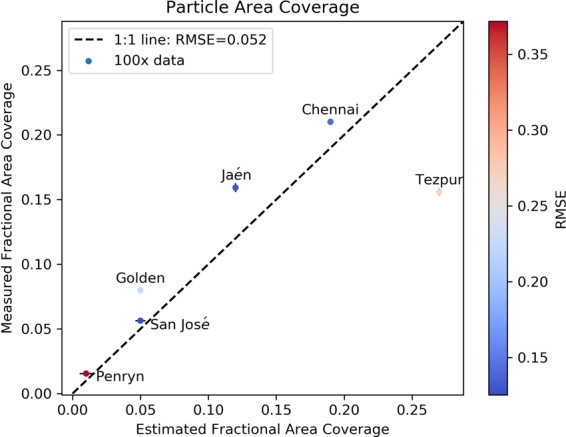
The fractional area coverage of the deposited particles measured directly using ImageJ (vertical axis) versus that estimated from Eq. (6) (horizontal axis). For this graph, all sites except Egypt were considered and only data obtained using the 100× magnification was utilized. The markers are colored according to the RMSE found in fitting Eq. (5).

The total fractional area coverage can be directly measured via the ImageJ analysis, for example as given from the peak of the cumulative fractional area coverage curve in Fig. 4 (right y-axis). These measured *f* values are collected in Table 3. Alternatively, *f* can be estimated by using the fits to the particle size distribution to obtain a cleanliness level (for example, from Fig. 5) that can then be utilized in Eq. (6). Figure 6 shows the *f* determined directly versus the *f* value obtained from the best-fit to IEST-STD-CC 1246E, resulting in an *L* value that can be used in Eq. (6). The results using six of the seven the soiled coupons do not correlate perfectly, but do support an approximately linear relationship with a slope of 1.0 for natural soiling.

The outlying data point is for Tezpur, India. This coupon was challenging in two regards: both replicates were scratched after dust accumulation; and the soiling was very non-uniform on length scales of several hundred micrometers (For the micrograph image for Tezpur, refer to Supplemental Information.). It exhibited an irregular and mottled pattern of particle clustering, and even some branched features that resemble fungal hyphae previously reported in other PV glass soiling studies^39–41^.

Another challenge concerned the sample from Egypt. It was found to be very non-uniform, and so a reliable particle count could not be extracted with ImageJ. It exhibited layers of sand particles on top of other layers. No other soiled glass coupon had such a complex morphology. There were quite harsh weather conditions during the soiling deposition, such as strong, sandy winds and a heavy rain. For future studies, therefore, the time period for the collection of soiling on glass coupons from places like Egypt should be shortened, perhaps to only one week, so that only a single layer is present.

Overall, our results imply that the particle size distribution can, to first order, ignore the composition of the deposits. To our knowledge, this is the first time it is shown that the distribution described in IEST-STD-CC 1246 cleanliness standard is also valid for soiling deposited outdoors. That standard was introduced to describe the cleanliness of contamination-critical products, such as clean rooms and spacecraft.

### Fractional area coverage vs. transmittance

We can combine the results from the two types of optical instruments, the spectrophotometer and the microscope. It is expected that the measured *f* should be closely related to the broadband hemispherical transmittance, τ_b_, since, to first order, the deposited particles block or obscure the passage of light through the glass to the solar cell. This is supported by the results from other studies^25,36,41^. The results of Tables 1 and 3 can be connected by realizing that 1 − τ_b_ represents the optical losses due to the presence of soiling.

**Figure 7:**
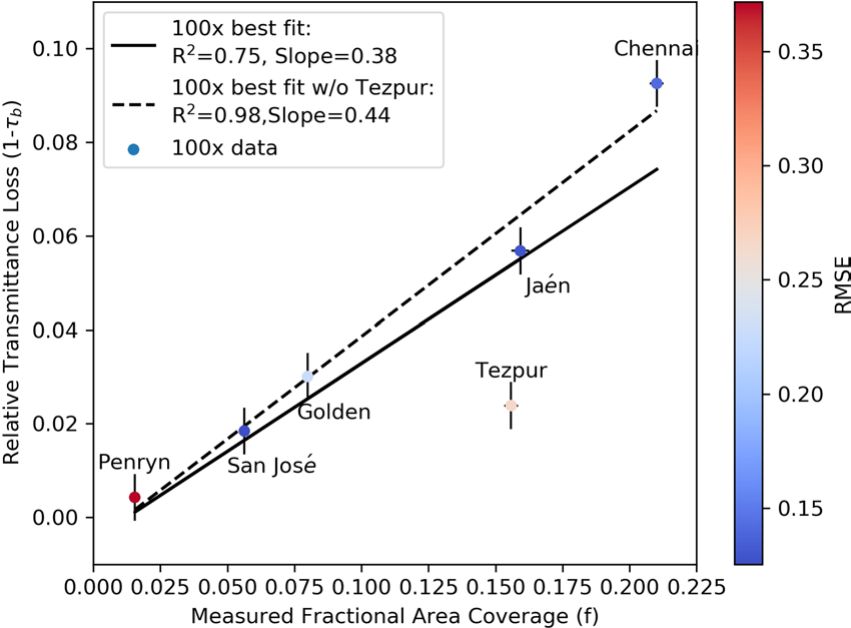
Broadband relative transmittance losses 1 − *τ*_b_ versus measured fractional area coverage *f* for all sites except for Egypt. The wavelength range for the transmittance was 350–1100 nm, while *f* is obtained from the 100× images using ImageJ. The markers are colored according to the RMSE found in fitting Eq. (5).

Figure 7 shows the relationship between this optical loss and the measured fractional area coverage by the dust particles (for example, from the cumulative fractional coverage of Fig. 4). While there is indeed a strong correlation between the two parameters, it does not have a slope of 1. This is not unexpected, because the particles are not all opaque, so some of the area covered by the particles continues to partially contribute to the transmitted radiation.

Better results were found for 100×, than at 500× (not shown) for almost all the sites, perhaps again because 100× is less susceptible to non-uniformity over the larger area viewed by the microscope. One should note that the area probed using the spectrophotometer is on the order of a square centimeter, while that for the images taken at 100× is on the order of a square millimeter. One way to mitigate this problem is to stitch together a tiled mosaic of microscope images so that they represent a larger area, as has been done in recent studies^36^.

The outlying data point in Fig. 7 was again for Tezpur, India. This was not unexpected, given the rationale describe previously. Taken together, however, the results of Figs. 5–7 suggest that we can estimate the transmittance of a PV cover glass from the measured fractional area coverage of deposited particles, and we can also estimate the impact of the spectral characteristics due to soiling from the size distribution and the cleanliness value, *L*. This relationship can find immediate application in monitoring PV soiling and estimating its effect on PV performance. Outdoor microscopes have already been employed to measure particle size and deposition rates related to soiling^42^, and could be used for the optical characterization of natural soiling of PV modules suggested by our work. The results augment and extend those of other studies made at lower soiling (i.e., lower *f* values)^43,44^.

### Utilizing the results

There are additional complexities for PV soiling that can now be discussed. Referring back to Fig. 1, one should recognize that AR or AS coatings will likely affect the smaller or larger particle sizes differently. The soiling ratio, the shape of the relative transmittance spectra and the fit to IEST-STD-CC 1246 standard will all be altered by coatings. Future work should therefore examine the spectral characteristics of the transmittance and the particle size distribution for coated and uncoated glass under the same environmental conditions. The effectiveness and efficacy of such coatings can be thus studied and characterized utilizing the techniques described in this report.

One should recognize that the composition of atmospheric particulates also depends on location and it varies with time. The composition of particles deposited by soiling therefore also varies. One of the main findings of this report is the fact that soiling collected on PV glass in different locations has a similar spectral behavior that can be modeled using the equations inspired by the Ångström turbidity equation. While the composition of the particles is certainly involved in how they adhere to the glass^36^, their general optical characteristics and their particle size distribution after deposition may not strongly depend on the details the chemical nature of the particles. The application of the Ångström turbidity equation and the IEST-STD-CC 1246 standard should therefore be further tested to establish its usefulness in estimating the impact of soiling on the electrical performance of PV modules. For the optical aspects of PV soiling that include particle composition, the more rigorous approach of Piedra *et al*.^32,33^ can be employed, for example by utilizing the real and imaginary parts of the refractive index of the particles.

Our work, and the work of others^16,17^, asserts that the measured spectral transmittance of the soiled cover glass on a PV module is relevant to quantify the impact of soiling on a module’s performance. With the completion of the studies of this report, this can now be explored further via measurements of a module’s effective transmittance. To accomplish this, the external spectral quantum efficiency, EQE(*λ*), or spectral response, SR(λ)^45,46^, can be utilized. It can first be measured for a small soiled module and then that module can be cleaned. The ratio of SR(*λ*), soiled to clean, can then be directly compared to the relative transmittance versus wavelength for the case of the glass alone as described in this study. This work is at present ongoing and will be described in a future report. The results and techniques described in the present report therefore serve as a baseline and reference for that research.

## Conclusions

In this study, we characterized the soiling that was naturally deposited on low-iron glass used for PV modules. Accumulation was sampled at seven locations worldwide with very different climactic and environmental conditions. A somewhat surprising result is that the spectral characteristics of the soiling were, in general, remarkably similar at these locations, in that they exhibited lower transmittance in the UV and blue regions of the spectrum, and a gradual asymptotic increase towards the red and infrared regions. The general shape of the transmittance curve can be described via a modified form of the Ångström turbidity equation, which itself has long been found to be useful in describing the transmittance of the Earth’s atmosphere due to the presence of aerosols and particulate matter. This is the first time that an equation that describes the attenuation of light by aerosols suspended in the atmosphere has been proposed and applied to describe the wavelength dependence of the transmittance of light due to those particles that end up adhering to the glass surface. This could provide a useful characterization tool to measure soiling losses in deployed photovoltaic arrays for different types of PV modules by considering the incoming spectral irradiance and each PV module’s spectral response.

We also found that the distribution of particle sizes at the various sites closely follows the distribution represented by the IEST-STD-CC 1246E cleanliness standard. To our knowledge, this is the first time it has been shown that the distribution described in this standard is valid also for outdoor-deposited soiling relevant to solar conversion. The cleanliness level *L* of naturally soiled coupons after eight weeks of exposure was found to be between 600 and 1200 µm. The fractional area coverage *f* could be measured and estimated using a formalism related to the cleanliness standard. As suggested by other studies, the transmittance linearly correlates to *f*, thus linking the measurements from a suitably equipped spectrophotometer and a microscope. Future work should therefore explore additional connections between the IEST-STD-CC 1246E cleanliness standard and the Ångström turbidity equation analysis approach.

The empirical models we have presented, relating to both the spectral and the particle size distribution, can serve as a reference for future studies, as researchers will be able to use them to optically characterize the soiling accumulated on PV glass and PV modules in order to more easily model the expected electrical losses. A variety of sites should be studied worldwide, but with a larger sample size and more replicates at each site so that a full statistical and uncertainty analysis can be performed.

## Methods

### Experimental campaign

Seven 4 cm × 4 cm, 3 mm thick Diamant low-iron glass coupons from Saint-Gobain Glass were shipped to different sites worldwide. The seven locations (listed and described in Supplementary Information) were chosen to represent a large variety of soiling and environmental conditions. To maximize the collection of particles, the glass was mounted in a horizontal orientation. The full details of the testing procedure has been described elsewhere^25^. At the end of the 8-week collection campaign, the coupons that were never cleaned were sent to NREL and the University of Exeter for detailed analysis. Each coupon was placed in an individual case for shipping, which limited the loss of dust and avoided cross-contamination among the coupons during the transportation. A loop of tape placed at between the bottom of the glass and the bottom of the box prevented the former from moving within the box. The coupons, and the cases, were visually inspected at arrival to check for a loss of dust from the surface of the glass, which did not occur.

Glass coupons are a standard method for the analysis of soiling of photovoltaics and have been used in a number of research papers^11,39,41,42,47–51^. In addition, recently launched low-maintenance soiling detectors quantify the loss of PV modules through the optical analysis of the contamination accumulated on glass^52,53^. DustIQ, produced by Kipp&Zonen, measures the backward reflection of a soiled PV glass, whereas MARS, developed by Atonometrics, measures the brightness of the pixels of a camera placed under the glass. Both prototypes are being tested and have shown good results when their measurements are compared with the soiling loss experienced by PV modules^54,55^.

### Soiling loss

The most common metric to quantify the soiling loss is the soiling ratio. This metric, defined in the IEC 61724-1 standard^7^, expresses the ratio between the performance of a soiled PV device under outdoor conditions and the performance of the same PV device but without soiling. It has a value of 1 if no soiling is present on the PV surface, while it decreases while soiling accumulates. In the present work, the methodology described in prior work^11^ has been followed. The performance of the modules has been quantified though the short-circuit current and, therefore, the soiling ratio at any given time, *t*, has can be expressed as:

7 $${r}_{s}(t)=\frac{Is{c}_{soil}(t)}{Is{c}_{ref}(t)}$$

where *Isc*_soil_ and *Isc*_ref_ are the short-circuit currents of the soiled and the clean, reference PV device. These currents can be measured directly from the PV devices, or can be related to the solar spectral irradiance and solar cells spectral response according to the following equations^56^:

8 $$Is{c}_{ref}(t)={A}_{PV}{\int }_{{\lambda }_{1}}^{{\lambda }_{2}}{E}_{G}(\lambda ,t)\cdot SR(\lambda )\cdot d\lambda $$

9 $$Is{c}_{soil}(t)={A}_{PV}{\int }_{{\lambda }_{1}}^{{\lambda }_{2}}{E}_{G}(\lambda ,t)\cdot \tau (\lambda ,t)\cdot SR(\lambda )\cdot d\lambda $$

where *λ*_1_ and *λ*_2_ are the lower and upper limits of the PV absorption band. In this work, the optical spectrum has been limited to the wavelength range 350 to 1100 nm. *A*_*PV*_ is the active area of the PV devices (solar cells in a module). *E*_*G*_(*λ*, *t*) is the spectral irradiance. For the results shown in Table 1, the standard Air Mass 1.5 solar irradiance spectrum^27^ has been utilized even though the soiling ratio is operationally determined for solar noon. *SR*(*λ*) is the spectral response of the photovoltaic material. The results in Table 1 have been obtained considering a monocrystalline silicon (c-Si) cell, although the equations above are valid for different materials. *τ*(*λ*, *t*) is the relative spectral hemispherical transmittance due to the soiling. Referring to Fig. 1, it should be recognized that it is also a function of the angle of incidence. The product, $\tau (\lambda ,t)\cdot SR(\lambda )\,$ would be the effective *external* spectral response of a soiled module.

### Optical characterization

The spectral hemispherical transmittance of the soiled coupons after the 8-week outdoor exposure was taken using a Cary 500 spectrophotometer with an integrating sphere attachment at 1 nm steps between 300 nm and 1100 nm. For the processing of the broadband transmittance, only the hemispherical transmittance between 350 nm and 1100 nm was considered, because of the confounding factor of the absorption of the glass itself at wavelengths less than 350 nm. Three measurements were taken per coupon and averaged, then three more measurements were taken in a different location on the coupon and averaged to partially mitigate the non-uniformity of soiling. Only one set of transmittance measurements (i.e., one spot) were available for Chennai and Jaén. The transmittance of a clean reference coupon was taken at the start and the end of each set of measurements to check the consistency of the measurement and to obtain the relative hemispherical transmittance of soiling, *τ*(*λ*), calculated as a ratio of the hemispherical transmittance of each soiled coupon to the hemispherical transmittance of the unsoiled reference coupon.

We also applied an offset correction to all the measurements to correct for detector and filter change near 800 nm. The offset was calculated as the difference between the broadband transmittance between 790 nm and 799 nm and the broadband transmittance between 800 nm and 809 nm. Then, the offset correction to all the data for wavelengths *λ* ≥ 800 nm was applied. Examples are shown in Supplementary Information. Although a full uncertainty analysis has not been completed for the spectrophotometer work, the standard uncertainty associated with the repeatability of the transmittance measurements is estimated to be ±0.005.

### Image processing and particle size distribution measurements

Microscope images were captured at 100× and 500× magnification for the coupons soiled at each of the seven sites. The image of each coupon has been taken using a Keyence VHX-5000 microscope, at a resolution of 1200 pixels × 800 pixels. Particles less than 2.4 µm in diameter could not be counted at 100 × (4.5 µm^2^ is the smallest area, as that was the pixel area at that magnification). The image settings (brightness, contrast, texture, color, and lighting) were adjusted to capture images under optimal conditions^41^. The micrographs were analyzed using the image processing software package, ImageJ^34^. Its auto threshold function was used to generate 8-bit grayscale images. Some images presented an oversaturation on one or more of the corners. In order to process all the images in the most consistent way, only the central part of the image was used for counting particles, using a rectangular mask.

ImageJ was used to count the particles and measure their area projected on the plane of the glass coupon. The area of each particle is given as *A*(*x*), where x is a counting index. The particle areas thus obtained were summed and the result was divided by the total analyzed area, *A*_*m*_, in the micrograph (4,536,862 µm^2^ for 100× and 166,667 µm^2^ for 500×) to yield the measured fractional area coverage, *f*. The fractional area coverage is given by,

10 $$f=\frac{1}{{A}_{m}}\sum _{x}A(x)$$

The results were further processed to estimate the effective particle diameter, *D*, and the particle size distribution, both the size distribution density and the cumulative values, *N*(*D*).

### Curve fitting

The curve fitting for Eqs. (2) and (3) was performed through the curve_fit function in the SciPy library for Python 2.7^57^. Nonlinear least-squares with a Trust Region Reflective algorithm were employed. The initial guesses and the boundary conditions for each variables are shown in Table 4. The regression of Eq. (5) has been conducted by determining the *L* value between 1 and 3000 (at steps of 1) that returned the lowest mean squared error.

**Table 4:** Initial guesses and boundary conditions for curve fitting of Eqs. (2) and (3).

	α	α*	β_sur_	β_sur_*	γ*
Initial Guess	1.75	1.75	0.001	0.001	−0.023
Boundary Conditions	0.0 to 10.0	0.0 to 10.0	0.0 to 0.5	0.0 to 0.5	None

### Uncertainties

An uncertainty of ±0.005 was considered in the measurement of the hemispherical transmittance. We also estimated an uncertainty in the estimation of the projected effective spherical particle diameter to be roughly 1 µm. This leads to uncertainties in the determination of *L* between 2% to 3% by repeating the analysis using particle diameters increased or decreased by 1 µm. To estimate the sampling size uncertainty, the ImageJ analysis was repeated with a rectangular mask of one half of the area used for the results reported. The resulting *L* values differed by 3% to 7%. Given the variance of the particle size distribution and that, with the exception of Penryn, the number of particles was over 3000, this uncertainty is reasonable^58^.

## Supplementary information

Supplementary Information.

## Data Availability

The datasets generated and analysed during the current study are available from the corresponding authors and in the Mendeley data repository, 10.17632/2pcpmp22fx.
