# Supplementary material for: Modelling photovoltaic soiling losses through optical characterization
Source: Sci Rep. 2020 Jan 9;10:58. doi: 10.1038/s41598-019-56868-z (PMC6952442; doi:10.1038/s41598-019-56868-z)
Supplement: Supplementary file 1 — Supplementary Information. [file 41598_2019_56868_MOESM1_ESM.pdf]

Supplementary Information (SI) for

Modelling photovoltaic soiling losses through optical characterization

Greg P. Smestad[1], Thomas A. Germer[2], Hameed Alrashidi[3], Eduardo F. Fernández[4], Sumon Dey[5], Honey Brahma[6], Nabin Sarmah[6], Aritra Ghosh[3], Nazmi Sellami[7,8], Ibrahim A. I. Hassan[9, 10], Mai Desouky[11], Amal Kasry[11], Bala Pesala[12], Senthilarasu Sundaram[3], Florencia Almonacid[4], K. S. Reddy[13], Tapas K. Mallick[3] & Leonardo Micheli[4,14]

[1]Sol Ideas Technology Development, P.O. Box 5729, San José, California, USA 95150. [2]National Institute of Standards and Technology, 100 Bureau Drive, Gaithersburg, MD, USA. [3]University of Exeter, Penryn, UK. [4]Centro de Estudios Avanzados en Energía y Medio Ambiente (CEAEMA), Universidad de Jaén, 23071, Jaén, Spain. [5]Academy of Scientific and Innovative Research, Chennai, India, 600113. [6]Tezpur University, Tezpur, India, [7]Heriot-Watt University, Dubai, UAE. [8]Robert Gordon University, Aberdeen, UK. [9]Department of Chemistry, South Valley University, 83523 Qena, Egypt. [10]Faculty of Environmental Studies, University of York, Toronto, M3J 1P3, ON, Canada. [11]Nanotechnology Research Centre, The British University in Egypt, 11837, El Sherouk City, Cairo, Egypt. [12]CSIR-Central Electronics Engineering Research Institute & Academy of Scientific and Innovative Research, Chennai, India, 600113. [13]Indian Institute of Technology Madras, Chennai, India. [14]National Renewable Energy Laboratory, Golden (CO), USA.

Correspondence and requests for materials should be addressed to G.P.S. (email: inquiries@solideas.com) or L.M. (email: lmicheli@ujaen.es).

Locations. The coupons were mounted for an 8-week period (January to March 2016) at the seven locations listed in **Table S1**. The samples were mounted horizontally (without a tilt). The sites include five countries (Egypt, India, Spain, UK, USA), that in 2018 were among the 20 countries with the largest PV capacity worldwide, or with the highest expected growth rate in installed PV, or both[1].

Chennai is the capital of Tamil Nadu state and sixth most populous city in India. Chennai is a coastal city and experiences hot and humid climate for most of the year. Chennai records its highest average temperature in May (~33 °C) and its lowest average temperature in January (~24.3 °C). Chennai receives rainfall during the period of August to November and is relatively dry during the rest of the year. It is among the highest polluted cities in India, and most of that is from automobiles. The coupons were installed on the roof of CSIR-CEERI, which is located in the center of the city.

El Shorouk is a city located at 40 km from Cairo, in Egypt. The area is basically a suburb town quite outside the city, with lots of residential compounds. It is located in a desert environment, exposed to strong winds and rain, especially in the winter months. The samples were mounted in a terrace inside the campus at the British University in Egypt.

Golden is a town of ~20,000 inhabitants located 20 km from Denver, Colorado, in the USA. It lies at an elevation of more than 1700 meters at the foot of the Rocky Mountains. The coupons were installed on the NREL campus at ground level in an area with limited traffic.

Jaén is a medium-size town located in southern Spain. It experiences low-medium values of airborne particulate matter, even if this can periodically reach unusually high values due to specific events. The province of Jaén is the largest producer of olive oil worldwide, and the activities of harvesting and of burning olive tree branches are major sources of soiling. Also, the region is exposed to occasional Saharan dust storms. The coupons were installed on the roof of the A3-building of the university, which is located on the edge of the city.

Penryn is a small coastal town in the west of Cornwall, the region with the highest irradiation in the UK. It is exposed to frequent, light rainfalls and high winds. Most of the precipitation occurs between September and March. The coupons were installed on the second floor terrace of the Environment and Sustainability Institute of the University of Exeter.

This is made available under the CC-BY-NC-ND 4.0 license creativecommons.org/licenses/by-nc-nd/4.0/

San José is the third most populated city in California and lies within the San Francisco Bay Area, the twelfth most populated metropolitan area of the USA. It is located about 42 km from the Pacific Ocean and is subject to seasonal rainfall (primarily from November to May). The coupons were installed on the roof of the first floor at Sol Ideas Technology Development.

Tezpur is a city located in Assam, India in the north-eastern part of India, at the bank of the river Brahmaputra near the Himalayan foothills. The climatic condition is normally warm and humid, except for a couple of the winter months. The location receives large amount of rainfall frequently during monsoon season and goes dry during winter season. The glass coupons were installed in the garden area near the roadside within the Tezpur University campus, which is situated in a rural area.

This is made available under the CC-BY-NC-ND 4.0 license creativecommons.org/licenses/by-nc-nd/4.0/

City	Coordinates	Climate Classification	Global Irradiance at optimum tilt angle [kWh/m2/year]	Yearly Rainfall Intensity [mm/year] (typical rainfall Intensity during data collection period [mm/year])	Annual average PM2.5 concentration in 2015 [μg/m^3]
Chennai, India	13.08N, 80.27E	Equatorial savannah with dry winter (Aw)	2009	1197 (20)	29.1
El Shorouk, Egypt	30.12N 31.61E	Desert climate (Bwh)	2332	18 (10)	67.1
Golden (CO), USA	39.74N 105.18W	Snow climate, fully humid (Dfb)	2050	420 (64)	9.2
Jaén, Spain	37.79N 3.78W	Warm temperate climate with dry summer (Csa)	2126	552 (222)	13.1
Penryn, UK	50.17N 5.13W	Warm temperate climate, fully humid (Cfb)	1221	1058 (316)	7.5
San José (CA), USA	37.29N 121.91W	Warm temperate climate with dry summer (Csb)	2213	385 (201)	11.3
Tezpur, India	26.70N 92.83E	Warm temperature climate with dry winter (Cwa)	1718	1836 (96)	33.4

Table S1. List of monitored locations. Climate Classifications are sourced from the Köppen-Geiger climate classification method[2]. The Global Irradiance at optimum tilt angle is sourced from the Global Solar Atlas[3]. The typical monthly rainfall intensities have been sourced from the on-line climate data[4], which uses data collected between 1982 and 2012. The data collection period took place between January and March 2016. The average atmospheric particulate matter, PM2.5, concentrations are sourced from the 0.1° × 0.1° resolution 2015 satellite database[5].

This is made available under the CC-BY-NC-ND 4.0 license creativecommons.org/licenses/by-nc-nd/4.0/

City	Broadband Transmittance	Solar Weighted Transmittance	Predicted Soiling Ratio
Chennai, India	0.907	0.904	0.909
El Shorouk, Egypt	0.669	0.658	0.673
	0.671	0.66	0.675
Golden (CO), USA	0.966	0.964	0.966
	0.974	0.973	0.974
Jaén, Spain	0.943	0.941	0.945
Penryn, UK	0.995	0.995	0.996
	0.996	0.995	0.966
San José (CA), USA	0.978	0.976	0.978
	0.985	0.984	0.986
Tezpur, India	0.975	0.974	0.976
	0.977	0.976	0.977

Table S2. The full data set that is summarized in Table 1 of the main text. Soiling transmittance, solar weighted transmittance and predicted soiling ratio (r_S) for a monocrystalline silicon cell at the indicated sites in the wavelength range from 350 nm to 1100 nm. Measurements were performed at two closely spaced spots on each sample, except for Chennai and Jaén.

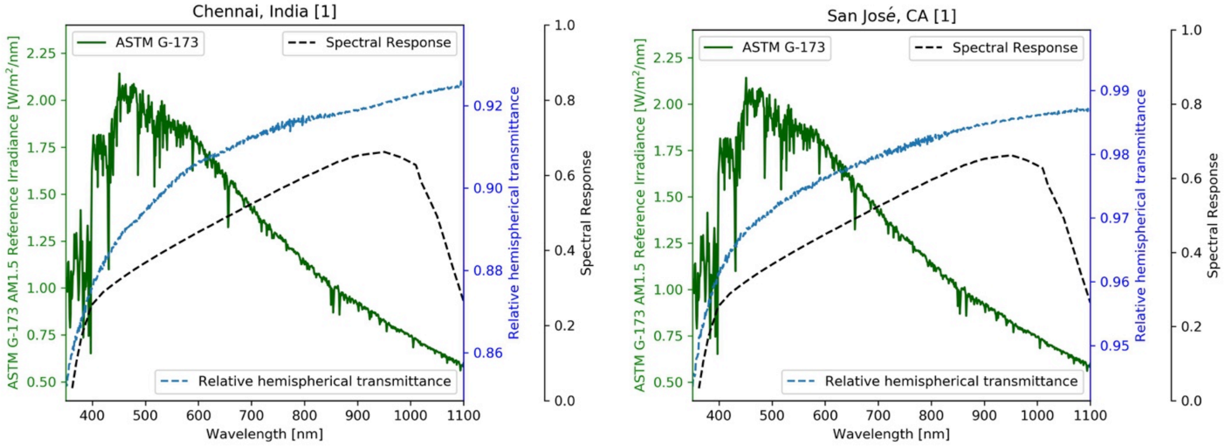

Figure S1. The hemispherical transmittance for two representative locations, together with the spectral response of a typical commercial monocrystalline Si solar cell and the ASTM G-173 AM1.5 reference irradiance[6]. Together with the equations found in the methods section of the paper, this data has been used in the calculation of the soiling ratio of **Table S2**.

This is made available under the CC-BY-NC-ND 4.0 license creativecommons.org/licenses/by-nc-nd/4.0/

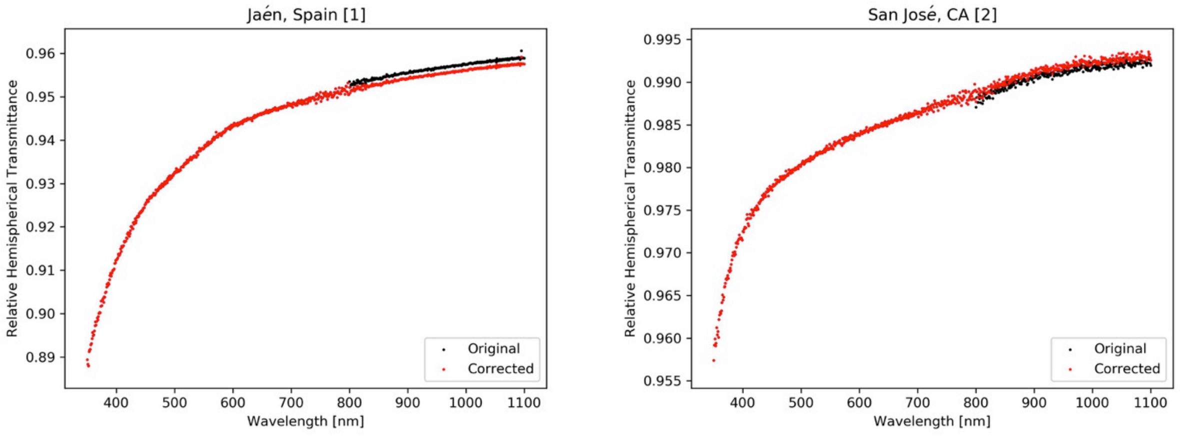

Figure S2. Examples of the offset corrections performed on the hemispherical transmittance. The title of each plot reports the location where the sample was soiled and the number of the measurement.

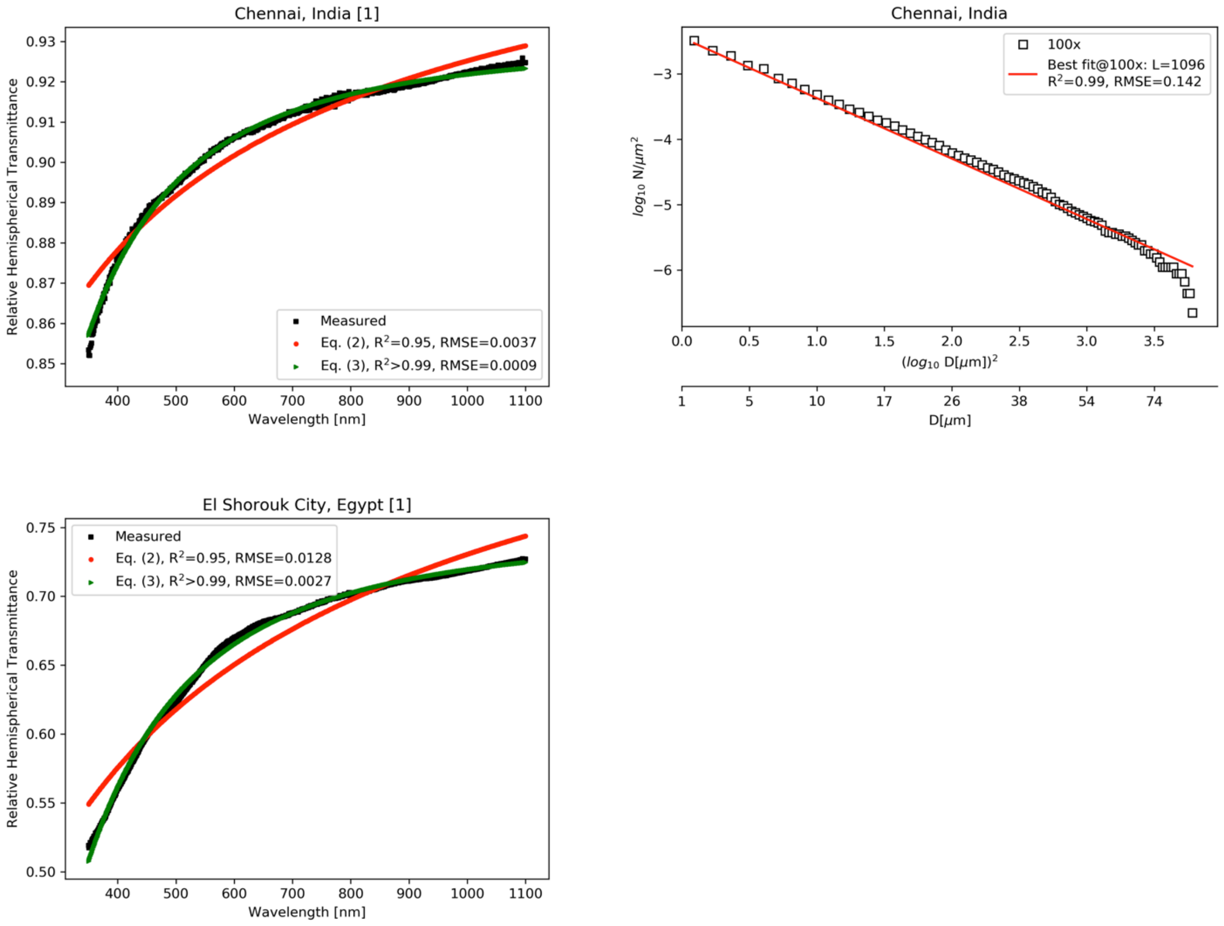

Figure S3. (left) The hemispherical transmittance vs. wavelength curves relative to that of clean glass. Also shown is the fit to the modified Ångström equations, (red) Eq. (2) and (green) Eq. (3); (right) Cumulative particle size distribution, N, and best fit to IEST-STD-CC 1246E at 100x for each location. The size distribution for Egypt could not be reliably obtained due to particle aggregation and layering.

This is made available under the CC-BY-NC-ND 4.0 license creativecommons.org/licenses/by-nc-nd/4.0/

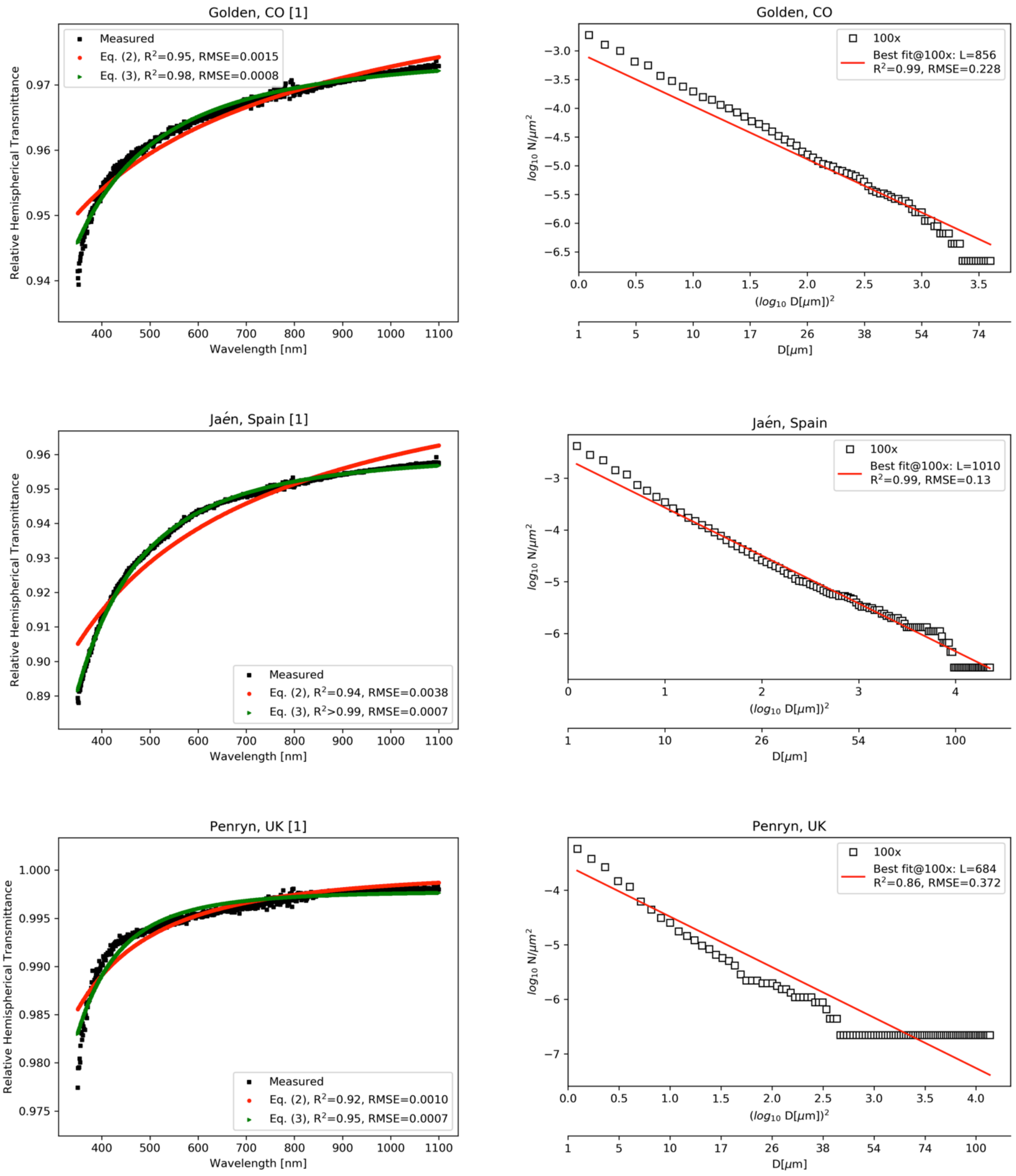

Figure S3. (left) The hemispherical transmittance vs. wavelength (350 nm to 1100 nm) curves for glass coupons soiled at each of the study locations. Also shown is the fit to the modified Ångström equations, (red) Eq. (2) and (green) Eq. (3). The measurements are relative to that of clean glass; (right) Cumulative particle size distribution, N, and best fit to IEST-STD-CC 1246E at 100x for each location.

This is made available under the CC-BY-NC-ND 4.0 license creativecommons.org/licenses/by-nc-nd/4.0/

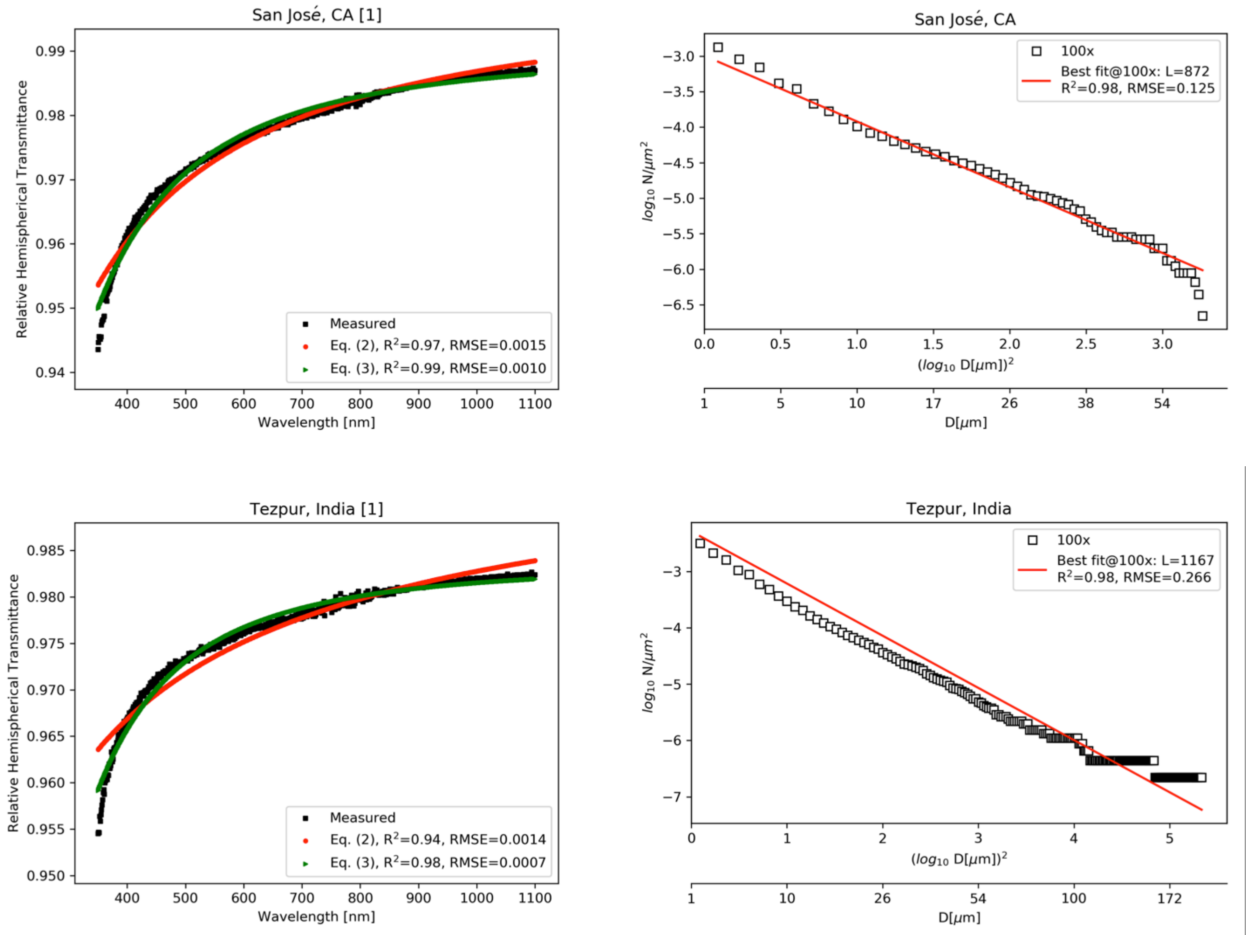

Figure S3. (left) The hemispherical transmittance vs. wavelength (350 nm to 1100 nm) curves for glass coupons soiled at each of the study locations. Also shown is the fit to the modified Ångström equations, (red) Eq. (2) and (green) Eq. (3). The measurements are relative to that of clean glass; (right) Cumulative particle size distribution, N, and best fit to IEST-STD-CC 1246E at 100x for each location.

This is made available under the CC-BY-NC-ND 4.0 license creativecommons.org/licenses/by-nc-nd/4.0/

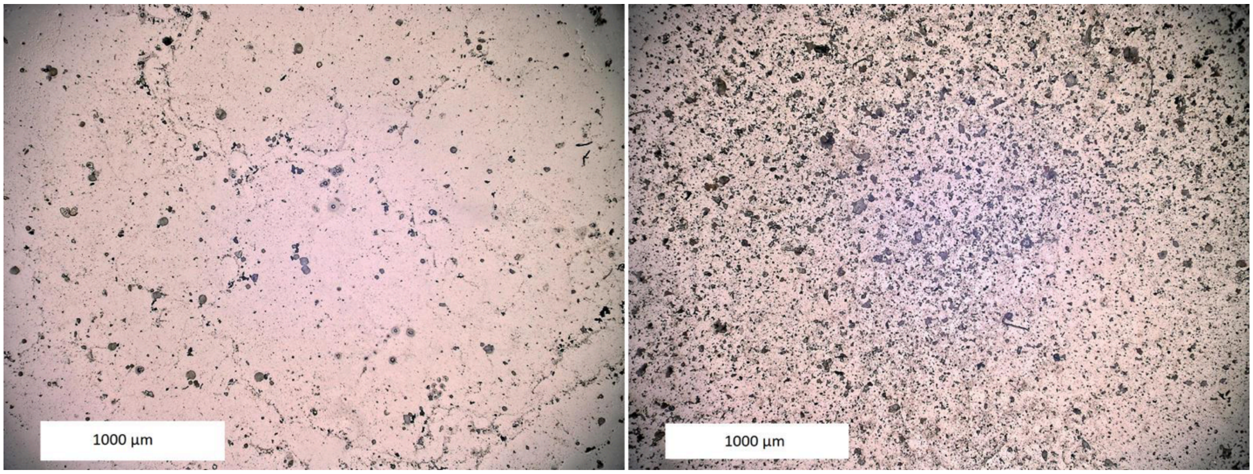

Figure S4. Representative images of soiled glass coupons from (left) Chennai and (right) San José at 100x magnification taken with a Keyence VHX-5000 microscope. The ImageJ processing program used micrographs such as these as input to estimate the effective particle diameter and the particle size

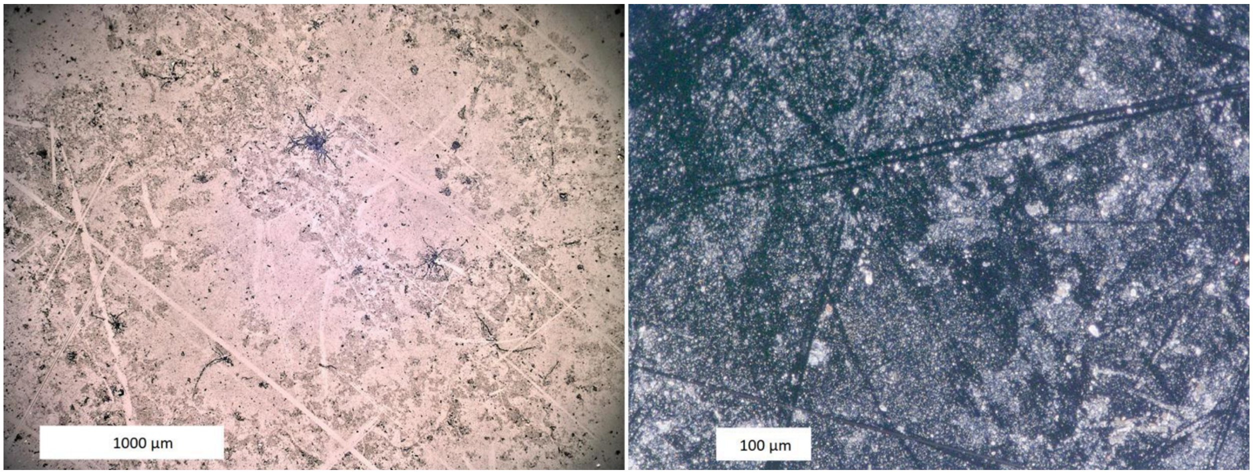

distribution for the coupons soiled at each of the seven sites.

Figure S5. Image taken with a Keyence VHX-5000 microscope of the soiled glass coupon from Tezpur, India, showing some scratches at (left) 100x and (right) at 500x. The image on the right shows a large number of very small particles. The scale bars are (left) 1000 µm and (right) 100 µm.

References

1. Schmela, M., Beauvais, A., Chevillard, N., Paredes, M.G., Heisz, M., Rossi, R., 2019. Global Market Outlook For Solar Power / 2019 - 2023.

2. Kottek, M., Grieser, J., Beck, C., Rudolf, B. & Rubel, F. World map of the Köppen-Geiger climate classification updated. *Meteorol. Zeitschrift* **15**, 259–263 (2006).

3. World Bank Group, Global solar atlas. Available at https://globalsolaratlas.info (Accessed: 15th September 2019).

4. Merkel, A. Climate data for cities worldwide [Data set]. AM Online Projects. Available at climate-data.org (2019).

This is made available under the CC-BY-NC-ND 4.0 license creativecommons.org/licenses/by-nc-nd/4.0/

5. Donkelaar, A. Van *et al.* Global Estimates of Fine Particulate Matter using a combined geophysical-statistical method with information from satellites , models, and monitors. *Environ. Sci. Technol.* **50**, 3762–3772 (2016).

6. NREL. Reference Solar Spectral Irradiance: Air Mass 1.5 [Data set]. Available at https://rredc.nrel.gov/solar//spectra/am1.5/ (2019).

This is made available under the CC-BY-NC-ND 4.0 license creativecommons.org/licenses/by-nc-nd/4.0/
